# The Test Your Memory cognitive screening tool: sociodemographic and cardiometabolic risk correlates in a population‐based study of older British men

**DOI:** 10.1002/gps.4377

**Published:** 2015-10-21

**Authors:** Efstathios Papachristou, Sheena E. Ramsay, Olia Papacosta, Lucy T. Lennon, Steve Iliffe, Peter H. Whincup, S. Goya Wannamethee

**Affiliations:** ^1^ Department of Primary Care and Population HealthUCLLondonUK; ^2^ Population Health Research InstituteSt George's, University of LondonLondonUK

**Keywords:** cognition, TYM, ageing, cognitive impairments

## Abstract

**Objective:**

This study aimed to examine the association of Test Your Memory (TYM)‐defined cognitive impairment groups with known sociodemographic and cardiometabolic correlates of cognitive impairment in a population‐based study of older adults.

**Methods:**

Participants were members of the British Regional Heart Study, a cohort across 24 British towns initiated in 1978–1980. Data stemmed from 1570 British men examined in 2010–2012, aged 71–92 years. Sociodemographic and cardiometabolic factors were compared between participants defined as having TYM scores in the normal cognitive ageing, mild cognitive impairment (MCI) and severe cognitive impairment (SCI) groups, defined as ≥46 (45 if ≥80 years of age), ≥33 and <33, respectively.

**Results:**

Among 1570 men, 636 (41%) were classified in the MCI and 133 (8%) in the SCI groups. Compared with participants in the normal cognitive ageing category, individuals with SCI were characterized primarily by lower socio‐economic position (odds ratio (OR) = 6.15, 95% confidence interval (CI) 4.00–9.46), slower average walking speed (OR = 3.36, 95% CI 2.21–5.10), mobility problems (OR = 4.61, 95% CI 3.04–6.97), poorer self‐reported overall health (OR = 2.63, 95% CI 1.79–3.87), obesity (OR = 2.59, 95% CI 1.72–3.91) and impaired lung function (OR = 2.25, 95% CI 1.47–3.45). A similar albeit slightly weaker pattern was observed for participants with MCI.

**Conclusion:**

Sociodemographic and lifestyle factors as well as adiposity measures, lung function and poor overall health are associated with cognitive impairments in late life. The correlates of cognitive abilities in the MCI and SCI groups, as defined by the TYM, resemble the risk profile for MCI and Alzheimer's disease outlined in current epidemiological models. © 2016 The Authors. *International Journal of Geriatric Psychiatry* Published by John Wiley & Sons, Ltd.

## Introduction

Current global estimates suggest that 25–35 million people are currently affected by severe cognitive impairments (SCI) (Qiu *et al*., 2009; World Health Organization, 2012), the most common form being Alzheimer's disease (ad) (Plassman *et al*., 2007). Established correlates for AD or even milder declines in cognitive abilities in late life include low physical activity (Winchester *et al*., 2013), impaired motor (Mirelman *et al*., 2014) and lung function (Bozek and Jarzab, 2011), and smoking (Anstey *et al*., 2007; Peters *et al*., 2008) as well as a positive history of cardiovascular diseases (CVD) (de la Torre, 2004) and/or diabetes (Tolppanen *et al*., 2013). Patients with cognitive impairments are also more likely to report poorer overall health (Montlahuc *et al*., 2011), sleep disturbances (Ownby *et al*., 2014) and higher levels of functional dependence (Aguero‐Torres *et al*., 1998). However, it remains unclear whether AD is associated with late‐life hypertension (Power *et al*., 2011), hypercholesterolaemia (Polidori *et al*., 2012) and obesity (Anstey *et al*., 2011).

The past four decades have seen growing interest in developing easy‐to‐administer cognitive screening tools with clinical utility for primary and secondary care settings (Larner, 2013; Zygouris and Tsolaki, 2014). One of the most promising tools is the Test Your Memory (TYM) cognitive test (Brown *et al*., 2009), which has sound psychometric properties (Brown *et al*., 2009; Hancock and Larner, 2011), remarkable cross‐cultural validity (Hanyu *et al*., 2011; Abd‐Al‐Atty *et al*., 2012; Szczesniak *et al*., 2013; Ferrero‐Arias and Turrion‐Rojo, 2014; Iatraki *et al*., 2014; Muñoz‐Neira *et al*., 2014; Postel‐Vinay *et al*., 2014) and good concurrent validity with established tests, such as the Mini‐mental state examination, and the Addenbrooke's cognitive examination test battery (Brown *et al*., 2009; van Schalkwyk *et al*., 2012; Koekkoek *et al*., 2013). However, studies using the TYM were conducted among small clinical samples and did not report on the sociodemographic and cardiometabolic correlates of TYM‐defined cognitive groups.

In this study, we investigated whether the established risk pattern for late‐life mild cognitive impairments (MCI) and SCI is the same in a general population sample of British men aged 71–92 years assessed with the TYM. In particular, we sought (a) to characterize the cognitive profiles defined by the TYM in terms of their sociodemographic and cardiometabolic correlates and (b) to examine whether the sociodemographic and cardiometabolic profile of TYM‐defined cognitive impairments is similar to the ones reported in studies employing different classification criteria to define groups with MCI or SCI. Data included sociodemographic, lifestyle and overall health characteristics, health service use, adiposity measures, cardiovascular risk factors or diseases, diabetes and blood markers. It was hypothesized that these risk markers would be significantly associated with differing abilities of cognitive functioning as defined by the TYM, in line with the epidemiological models proposed for patients with MCI and SCI.

## Methods

**Figure 1:**
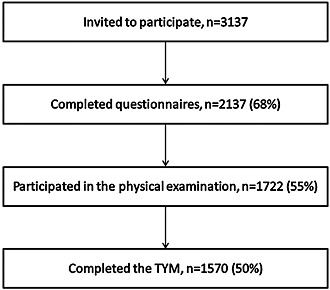
Recruitment and retention flow chart for the 30‐year replication of the British Regional Heart Study (2010–2012).

The British Regional Heart Study (BRHS) is a prospective study including a socially and geographically representative sample of 7735 men aged 40–59 years recruited from 24 towns representative of all major British regions. The BRHS commenced in 1978–1980 and did not cover the study of women (Walker *et al*., 2004). In 2010–2012, all surviving men (*n* = 3137) aged 71–92 years were invited to attend a 30‐year re‐examination (Figure 1). It was attended by 1722 BRHS participants (55% response rate). Ethical approval was provided by all relevant local research ethics committees. All men provided written informed consent to the investigations, which were carried out in accordance with the Declaration of Helsinki.

Physical examination of subjects involved anthropometric (waist circumference) and physiological (blood pressure, forced expiratory volume (FEV_1_) in 1 s) measurements as well as the calculation of body mass index (BMI). Physical performance assessments included a walking test, a chair rise test (time taken, in seconds, to sit down and stand up five times from a chair with the arms folded across the chest) and grip strength. The details of these assessments have been described elsewhere (Ramsay *et al*., 2014).

Cognitive skills were assessed using the TYM (Brown *et al*., 2009). The TYM is a 10‐task self‐assessment test that covers a broad range of cognitive domains including orientation, copying, semantic knowledge, calculation, fluency, similarities, naming, visuospatial abilities, anterograde memory and executive functioning (Brown *et al*., 2009). The last item is scored according to whether the TYM was completed with help from others or not (major = 1 to none = 5). Because participants completed the TYM in a controlled setting without assistance, the maximum score of 5 was given to all participants for this item. Total TYM scores range between 0 and 50 with higher scores indicating superior cognitive performance. Upon calculating the total TYM scores, participants were divided into three categories: a normal cognitive ageing group, a group with MCI and one with SCI. Cut‐offs for the respective categories were based on the original TYM scores (Brown *et al*., 2009; Royal College of Psychiatrists (RPsych)). Specifically, scores below 33 were considered consistent with SCI, while scores between 33 and 45 (if older than 80 years of age) or 46 (if younger than 80 years of age) were considered to be indicative of MCI.

Socio‐economic position was defined based on the longest‐held occupation of subjects at study entry (aged 40–59 years) in accordance to the Registrar Generals' Social Class Classification and has been described elsewhere (Wannamethee *et al*., 1996).

### Blood measurements

Assessments of high‐density lipoprotein cholesterol, triglycerides and glucose have been described in a previous study (Ramsay *et al*., 2014). Insulin resistance was estimated according to the homeostasis model assessment as the product of fasting glucose (mmol/l) and insulin (μU/ml) divided by the constant 22.5 (Ferrara and Goldberg, 2001).

### Lifestyle factors

Subjects were asked detailed questions about their smoking and drinking habits as well as their pattern of physical activity. These variables have been described in a previous study (Ramsay *et al*., 2014). Sleep quality was assessed using a self‐reported measure of the quality of sleep ranging from 1 ‘excellent’ to 4 ‘poor’ as well as a self‐reported measure of the average hours of the daytime and night‐time sleep. To assess social interactions, a modified version of the social engagement scale developed for the Nottingham Activity and Ageing Study (Harwood *et al*., 2000) was used; participants were asked whether they spend any time (a) with family, friends and neighbours, (b) with friends/relatives on the telephone, (c) in paid work, (d) in voluntary work, (e) in a pub or club, (f) attending religious services, (g) playing cards, games or bingo, (h) reading or (i) attending class or course of study. The sum of these questions ranged from 0 to 9 and scores ≤ 3 indicated low social interactions.

### Overall health, disabilities and health service use

History of cardiovascular disorders, that is, myocardial infarction, heart failure, stroke, angina and diabetes, was collected using information from the participants' general practitioner record reviews prior to their physical examination date. Participants were classified as having a disability if they reported difficulties in carrying out any of the following activities as a result of a long‐term health problem: going up or down stairs, bending down, straightening up, keeping balance, going out of the house or walking 400 yards. Mobility problems were defined as not being able to walk more than 200 yards, not being able to walk up and down a flight of 12 stairs without resting or not being able to bend down and pick up a shoe from the floor. Overall health was assessed using the number of general practitioner consultations in the last year, as well as using a self‐report health scale ranging from 0 (worst imaginable health state) to 100 (best imaginable health state). Depressive symptomatology was assessed using the four‐item Geriatric Depression Scale (D'Ath *et al*., 1994), a short instrument consisting of four questions based on the early work of Yesavage *et al*. (1982), which has been shown to have good sensitivity and specificity rates for the detection of depression in ill geriatric patients (Shah *et al*., 1997). Scores for this scale range from 0 to 4 with scores >2 being indicative of depression.

### Cut‐offs used for cardiometabolic characteristics

The definitions used to identify patients with hypertension as well as the cut‐offs for low levels of high‐density lipoprotein cholesterol and high levels of triglycerides have been described elsewhere (Ramsay *et al*., 2014). Impaired fasting glucose was taken as >6.1 and <7 mmol/l. Low FEV_1_ was defined as being in the lowest quintile of FEV_1_.

### Statistical analysis

Statistical analysis was conducted in four stages. First, the internal consistency of the items comprising the TYM was examined using Cronbach's alpha. Next, the proportion (%) of participants classified in each respective group (normal cognitive ageing, MCI or SCI) was computed. Third, the proportion (%) of participants who scored the maximum possible points in each TYM item across groups was computed to identify the affected cognitive domains. Finally, multiple logistic regressions were performed to estimate age‐adjusted odds ratios (ORs) and 95% confidence intervals (CI) for sociodemographic and cardiometabolic characteristics according to categories of cognitive impairment using ‘normal cognitive ageing’ as the reference group. The *p*‐value for linear trend was computed for all regression models and compared with a significance level adjusted for multiple comparisons (=0.002). Additional regression models were run for analyses examining the relationships between blood measures and cognitive functioning upon adjustment for adiposity measures because excess weight has been associated with insulin resistance and hypercholesterolaemia (Bagi, 2009). All analyses were carried out using stata/ic 13 (Stata Corp, College Station, TX, USA).

## Results

The analyses for this study were restricted to 1570 participants who completed the TYM. Items comprising the TYM presented with satisfactory internal consistency (Cronbach's *α* = 0.72).

Of the 1570 men aged 71–92 years, 801 (51%) had cognitive skills in the normal cognitive ageing range (*M* = 47.73, *SD* = 1.49), while 636 (41%) had MCI (*M* = 41.21, *SD* = 3.13) and 133 (8%) SCI (*M* = 27.43, *SD* = 5.33). Results of a one‐way analysis of variance suggested that the mean differences in the TYM scores were statistically significant between groups (*F*(2, 1567) = 3469.18, *p* < 0.001; *p*‐values of all pairwise comparisons <0.001). Table 1 summarizes the proportions (%) of participants who scored the maximum possible points for items assessing different cognitive domains. The results suggest that for all items comprising the TYM, higher rates of participants classified in the normal cognitive ageing group were able to obtain maximum scores (proportions of people scoring maximum points ranged from 55% to 78%), followed by those with MCI (22–39%) and SCI (0–6%).

**Table 1:** Overall cognitive performance and score per cognitive domain across cognitive performance groups defined using the TYM in a population‐based study of 1570 older British men aged 71–92 years

	**Normal cognitive ageing (*n* = 801, 51%)**	**Mild cognitive impairment (*n* = 636, 41%)**	**Severe cognitive impairments (*n* = 133, 8%)**	**Total sample (*n* = 1570)**
**Overall cognitive performance**				
Total TYM score (*M* ± *SD*)*	47.73 ± 1.49	41.21 ± 3.13	27.43 ± 5.33	43.37 ± 6.38
**Affected cognitive domains**				
**Orientation** *n* (%) with highest score (=10)	698 (58%)	438 (36%)	66 (5%)	1202 (77%)
**Copying** *n* (%) with highest score (=2)	778 (55%)	556 (39%)	77 (5%)	1411 (90%)
**Semantic knowledge** *n* (%) with highest score (=3)	574 (67%)	241 (28%)	37 (4%)	852 (54%)
**Calculation** *n* (%) with highest score (=4)	630 (58%)	399 (37%)	55 (5%)	1084 (69%)
**Fluency** *n* (%) with highest score (=4)	661 (69%)	276 (29%)	24 (3%)	961 (61%)
**Similarities** *n* (%) with highest score (=4)	653 (65%)	318 (32%)	29 (3%)	1000 (64%)
**Naming** *n* (%) with highest score (=5)	719 (57%)	497 (39%)	48 (4%)	1264 (81%)
**Visuospatial 1** *n* (%) with highest score (=3)	719 (62%)	418 (36%)	19 (2%)	1156 (74%)
**Visuospatial 2** *n* (%) with highest score (=4)	712 (63%)	390 (35%)	29 (3%)	1131 (72%)
**Anterograde memory** *n* (%) with highest score (=6)	504 (78%)	142 (22%)	1 (0%)	647 (41%)

TYM; Test Your Memory.

* *p* < 0.001.

Table 2 presents the age‐adjusted sociodemographic characteristics and lifestyle factors of the three cognitive function groups. Compared with participants in the normal cognitive ageing group, those with SCI were more likely to be of manual social class, physically inactive and ex‐smokers. In addition, they were more prone to report worse sleep quality, fewer hours of night‐time and more hours of daytime sleep as well as limited social interactions. With the exception of the duration of night‐time sleep, the relationships were also significant, albeit slightly weaker, for participants with MCI. *p*‐values for linear trend across ordered categories for these risk factors were statistically significant and survived adjustments for multiple comparisons, suggesting that risk exposure was systematically higher for participants in different cognitive groups (all *p*‐values <0.002).

**Table 2:** Sociodemographic and lifestyle factors across cognitive performance groups defined using the TYM in a population‐based study of 1570 older British men aged 71–92 years

	**Normal cognitive ageing (*n* = 801, 51%)**	**Mild cognitive impairment (*n* = 636, 41%)**	**Severe cognitive impairments** (*n* = 133, 8%)	***p*‐value** ‡ **(linear trend)**
**Age** (*M* ± *SD*) Odds ratio (95% CI)	78.09 ± 4.39 1.00	78.32 ± 4.69 1.01 (0.99–1.08)	78.88 ± 4.76 1.04 (1.00–1.08)	0.16
**Manual social class** *n* (%) Odds ratio (95% CI)	272 (35%) 1.00	334 (54%) 2.20 (1.77–2.74)**	100 (76%) 6.15 (4.00–9.45)**	<0.001
**Physical inactivity** *n* (%) Odds ratio (95% CI)	274 (36%) 1.00	247 (42%) 1.29 (1.03–1.61)**	61 (50%) 1.77 (1.20–2.61)**	0.001
**Smoking, never smoked** *n* (%) Odds ratio (95% CI)	344 (43%) 1.00	209 (33%) 0.66 (0.53–0.82)**	41 (31%) 0.61 (0.41–0.90)*	<0.001
**Alcohol consumption, moderate/heavy drinker** *n* (%) Odds ratio (95% CI)	25 (2%) 1.00	10 (2%) 0.85 (0.38–1.92)	3 (2%) 1.29 (0.37–4.53)	0.99
**Fair or poor sleep quality** *n* (%) Odds ratio (95% CI)	273 (35%) 1.00	237 (38%) 1.17 (0.94–1.45)	68 (53%) 2.09 (1.44–3.05)*	<0.001
**Hours of night‐time sleep,** Lower quintile (<6.0 h) *n* (%) Odds ratio (95% CI)	277 (35%) 1.00	249 (40%) 1.24 (1.00–1.54)	67 (52%) 1.95 (1.34–2.84)**	<0.001
**Hours of daytime sleep,** Top quintile (≥1.5 h) *n* (%) Odds ratio (95% CI)	48 (7%) 1.00	61 (12%) 1.74 (1.17–2.59)*	27 (26%) 4.46 (2.63–7.58)**	<0.001
**Limited social interactions** *n* (%) with 0–3 of 9 social interactions Odds ratio (95% CI)	78 (15%) 1.00	86 (25%) 1.86 (1.32–2.62)**	18 (28%) 2.11 (1.17–3.83)**	<0.001

All odds ratios are age‐adjusted.

TYM, Test Your Memory; CI, confidence interval.

* *p* < 0.05.

** *p* < 0.01.

‡ *p* for linear trend across ordered categories.

Table 3 presents the age‐adjusted associations between cognitive groups and history of CVD or diabetes, overall health, health service use, physical performance and disabilities. The results suggest that participants with SCI were more likely to have a positive history of stroke or diabetes or to report poorer overall health. Moreover, their average walking speed was slower; they performed poorly in the chair rise test, and they presented with decreased mobility and problems with keeping balance, as well as with other disabilities (all *p*‐values <0.001). Similarly, in comparison with participants in the normal cognitive ageing group, those with MCI were more likely to have a positive history of stroke and to report poorer overall health, have slower average walking speed and require longer times to complete the chair rise test. They were also more prone to have problems with keeping their balance and to have mobility problems and other disabilities. *p*‐values (trend across ordered groups) adjusted for multiple comparisons suggested that history of stroke or CVDs was marginally associated with cognitive groups (for both comparisons *p* = 0.003); trend *p*‐values for all other associations, including poor overall health, gait speed, problems with keeping balance, disabilities and mobility problems survived adjustments for multiple comparisons (<0.002)

**Table 3:** History of CVD, service use, overall health, disabilities and physical performance across cognitive performance groups defined using the TYM in a population‐based study of 1570 older British men aged 71–92 years

	**Normal cognitive ageing (*n* = 801, 51%)**	**Mild cognitive impairment (*n* = 636, 41%)**	**Severe cognitive impairments (*n* = 133, 8%)**	***p*‐value** ‡ **(linear trend)**
***History of CVD or diabetes***				
**Myocardial infarction** *n* (%) Odds ratio (95% CI)	69 (9%) 1.00	59 (9%) 1.09 (0.75–1.56)	16 (12%) 1.43 (0.80–2.55)	0.25
**Heart failure** *n* (%) Odds ratio (95% CI)	27 (3%) 1.00	34 (5%) 1.60 (0.95–2.69)	8 (6%) 1.74 (0.77–3.94)	0.05
**Stroke** *n* (%) Odds ratio (95% CI)	32 (4%) 1.00	44 (7%) 1.80 (1.13–2.87)*	12 (9%) 2.41 (1.21–4.81)*	0.003
**Diabetes** *n* (%) Odds ratio (95% CI)	91 (11%) 1.00	89 (14%) 1.29 (0.94–1.76)	23 (17%) 1.69 (1.03–2.80)*	0.03
**Any CVD** *n* (%) (MI, HF or stroke) Odds ratio (95% CI)	117 (15%) 1.00	118 (19%) 1.33 (1.00–1.76)*	32 (24%) 1.82 (1.17–2.84)**	0.003
***Service use and overall health***				
**Number of GP consultations in last year** Top quintile (≥6) *n* (%) Odds ratio (95% CI)	136 (18%) 1.00	116 (20%) 1.17 (0.88–1.54)	29 (24%) 1.44 (0.92–2.29)	0.08
**Poor overall health** *n* (%) Odds ratio (95% CI)	187 (24%) 1.00	188 (30%) 1.38 (1.09–1.75)**	59 (46%) 2.63 (1.79–3.87)**	<0.001
**Depressive symptomatology** *n* (%) with score >2 Odds ratio (95% CI)	47 (6%) 1.00	38 (7%) 1.11 (0.72–1.74)	8 (7%) 1.17 (0.54–2.55)	0.56
***Physical performance and disabilities***				
**Walking speed** (3 m) Top quintile (≥4.12 s) *n* (%) Odds ratio (95% CI)	116 (15%) 1.00	157 (25%) 1.93 (1.47–2.54)**	49 (37%) 3.34 (2.20–5.07)**	<0.001
**Sit/stand five times** Top quintile (≥17.54 s) *n* (%) Odds ratio (95% CI)	154 (19%) 1.00	178 (28%) 1.62 (1.26–2.08)**	35 (26%) 1.41 (0.91–2.17)	0.001
**Grip strength** Lower quintile (<23) *n* (%) Odds ratio (95% CI)	149 (19%) 1.00	139 (23%) 1.21 (0.93–1.57)	26 (20%) 0.97 (0.61–1.56)	0.30
**Problem keeping balance** *n* (%) Odds ratio (95% CI)	87 (14%) 1.00	87 (19%) 1.43 (1.02–1.98)*	28 (29%) 2.47 (1.49–4.10)**	<0.001
**Any disability** *n* (%) Odds ratio (95% CI)	222 (34%) 1.00	221 (44%) 1.49 (1.17–1.90)**	64 (55%) 2.30 (1.53–3.43)**	<0.001
**Mobility problems** *n* (%) Odds ratio (95% CI)	116 (15%) 1.00	143 (24%) 1.81 (1.38–2.39)**	55 (45%) 4.61 (3.04–6.97)**	<0.001

All odds ratios are age‐adjusted.

CVD, cardiovascular diseases; GP, general practitioner; MI, myocardial infarction; HF, heart failure; TYM, Test Your Memory; CI, confidence interval.

* *p* < 0.05.

** *p* < 0.01.

‡ *p* for linear trend across ordered categories.

Finally, Table 4 presents the age‐adjusted comparisons of blood markers, blood pressure, adiposity measures and lung function in the three cognitive function groups. Initial results showed that low high‐density lipoprotein and high insulin resistance were associated with a higher probability of being in the SCI group. However, after adjusting for BMI, the ORs associated with these blood markers were no longer statistically significant (OR = 1.38, 95% CI 0.82–2.33; and OR = 1.39, 95% CI 0.85–2.27, respectively). In contrast, obesity (BMI > 30 kg/m^2^) and high waist circumference (>102 cm) were significantly associated with SCI. Additionally, participants with SCI were more likely to have lower FEV_1_ than those in the normal cognitive ageing group. High blood pressure and other blood markers were not significantly associated with cognitive function group. Compared with participants in the normal cognitive ageing group, those with MCI did not differ significantly in any of the examined cardiometabolic risk factors or adiposity measures. *p*‐values (trend) adjusted for multiple comparisons suggested that a BMI > 30, waist circumference > 102 and membership in the bottom quintile of FEV_1_ showed a statistically significant trend across the different TYM‐defined cognitive groups (all *p*‐values < 0.002).

**Table 4:** Cardiometabolic risk factors, adiposity measures and lung function across cognitive performance groups defined using the TYM in a population‐based study of 1570 older British men aged 71–92 years

	**Normal cognitive ageing (*n* = 801, 51%)**	**Mild cognitive impairment (*n* = 636, 41%)**	**Severe cognitive impairments (*n* = 133, 8%)**	***p*‐value** ‡ **(linear trend)**
***Cardiometabolic risk factors***				
**Low HDL** (<1.04 mmol/l) *n* (%) Odds ratio (95% CI)	92 (12%) 1.00	93 (15%) 1.31 (0.96–1.79)	24 (19%) 1.76 (1.07–2.89)*	0.01
**High LDL** (>4 mmol/l) *n* (%) Odds ratio (95% CI)	65 (9%) 1.00	52 (9%) 1.01 (0.69–1.47)	8 (7%) 0.76 (0.35–1.62)	0.60
**High triglycerides** (≥2.3 mmol/l) *n* (%) Odds ratio (95% CI)	53 (7%) 1.00	53 (9%) 1.30 (0.87–1.93)	11 (9%) 1.40 (0.71–2.77)	0.23
**Total cholesterol** (≥5 mmol/l) *n* (%) Odds ratio (95% CI)	306 (40%) 1.00	216 (36%) 0.82 (0.66–1.03)	38 (31%) 0.67 (0.44–1.01)	0.02
**Impaired total fasting glucose** (<6.1 or >7.0 mmol/l) *n* (%) Odds ratio (95% CI)	73 (10%) 1.00	69 (12%) 1.18 (0.83–1.68)	11 (10%) 0.90 (0.46–1.76)	0.70
**Insulin resistance**, top quintile (≥3.68 mmol/l × μU/ml) *n* (%) Odds ratio (95% CI)	126 (18%) 1.00	116 (20%) 1.17 (0.88–1.55)	34 (30%) 1.97 (1.26–3.08)**	0.01
**High blood pressure** *n* (%) Odds ratio (95% CI)	545 (68%) 1.00	429 (68%) 0.97 (0.78–1.22)	92 (69%) 1.04 (0.70–1.55)	0.97
***Adiposity measures and lung function***				
**BMI** ≥ 30 kg/m^2^ *n* (%) Odds ratio (95% CI)	137 (17%) 1.00	124 (20%) 1.19 (0.91–1.56)	44 (34%) 2.59 (1.72–3.91)**	<0.001
**High waist circumference** (>102 cm) *n* (%) Odds ratio (95% CI)	288 (36%) 1.00	259 (41%) 1.23 (1.00–1.53)	69 (52%) 1.98 (1.36–2.87)**	0.001
**FEV_1_** Bottom quintile (≤1.93 l) *n* (%) Odds ratio (95% CI)	139 (18%) 1.00	132 (22%) 1.25 (0.95–1.64)	42 (33%) 2.24 (1.46–3.43)**	<0.001

All odds ratios are age‐adjusted.

HDL, high‐density lipoprotein; LDL, low‐density lipoprotein; TYM, Test Your Memory; CI, confidence interval.

* *p* < 0.05.

** *p* < 0.01.

‡ *p* for linear trend across ordered categories.

## Discussion

This study in a representative sample of older British men shows that numerous sociodemographic and cardiometabolic factors are significantly associated with MCI and SCI, as assessed using the TYM. The strongest associations were observed for factors that have also been associated with MCI and AD in different studies and included low occupation‐based socio‐economic status (Karp *et al*., 2004), physical inactivity (Winchester *et al*., 2013), motor (Mirelman *et al*., 2014) and lung dysfunction (Bozek and Jarzab, 2011), smoking (Anstey *et al*., 2007; Peters *et al*., 2008), positive history of CVD (de la Torre, 2004) or diabetes (Tolppanen *et al*., 2013), poor overall health (Montlahuc *et al*., 2011), sleep disturbances (Ownby *et al*., 2014) and higher levels of functional dependence related to abilities to perform everyday activities (Aguero‐Torres *et al*., 1998; Femminella *et al*., 2014). These correlates showed a significant ordered trend across cognitive groups suggesting a systematically increased likelihood of presenting with MCI and SCI.

### Comparison with other studies

While some of the significant correlates of cognitive functioning assessed with the TYM were consistent with findings from the available literature, late‐life hypertension (Power *et al*., 2011) and hypercholesterolaemia (Polidori *et al*., 2012), which have shown contradictory results with respect to incident AD, did not emerge as significant characteristics in this study. In addition, obesity was one of the strongest correlates of SCI although a recent meta‐analysis suggests that primarily midlife rather than late‐life obesity is associated with cognitive impairments (Anstey *et al*., 2011). However, the cross‐sectional design of this study did not allow for the examination of the prospective relationships between these measures. Moreover, lung function and adiposity measures were associated with SCI but not MCI and might therefore serve as target factors in future research on the differing manifestations and progress of MCI and AD. Two studies by Scarmeas *et al*. (2009a, 2009b) in prospective cohort samples of elders have also shown that BMI differs significantly only between individuals with intact cognitive skills and SCI, but not MCI (Scarmeas *et al*., 2009a, 2009b). With respect to lung function, reduced FEV_1_ rates in midlife were shown to be predictive of MCI in a recent study (Vidal *et al*., 2013), while declines in pulmonary function over an 8‐year period of time were not. The biological mechanisms underlying these relationships are yet to be disentangled.

### Strengths and limitations

This study is unique in terms of using the TYM in a general population sample to assess cognitive abilities in the elderly population. Previous studies using the TYM were based on clinical or smaller probabilistic samples and focused mainly on the discriminant abilities of the TYM rather than the characteristics associated with the cognitive groups assessed. Moreover, for this study, we assessed numerous sociodemographic and cardiometabolic factors, and therefore, it is an excellent inventory of cardiovascular and related factors associated with cognitive impairments.

The items of the TYM showed satisfactory internal consistency (72%) in this general population sample. While reliability rates between 78% and 98% have been reported for the TYM in clinical samples (Abd‐Al‐Atty *et al*., 2012; Muñoz‐Neira *et al*., 2014), Charter (2003) has noted that with low sample sizes, alpha coefficients can be rather unstable (Charter, 2003); the reported reliability of 0.72 might therefore represent a more accurate estimate.

The BRHS is a highly representative sample of the older male UK population, which has been successful in keeping attrition rates at very low levels. However, the issue of survivor bias cannot be overlooked in cohorts of ageing populations; subjects with AD are more likely to have died earlier (Weuve *et al*., 2014). An additional limitation of the BRHS is that it comprises only men of predominantly White European origin; therefore, the results cannot necessarily be generalized to women and different ethnic groups.

For this study, total TYM scores were calculated, which may have obscured findings on atypical, for example non‐amnestic, presentations of SCI. In fact, the TYM includes items assessing visuospatial tasks, which contribute 7/50 points and could help in distinguishing between amnestic and non‐amnestic (atypical) manifestations of MCI or SCI. However, non‐amnestic AD is less frequent than amnestic AD (Chertkow *et al*., 2013). Additionally, the TYM is not a diagnostic tool, and therefore, it is likely that study members with SCI did not meet diagnostic criteria for AD or suffered from other forms of dementia.

It is also likely that different cut‐offs of the TYM to define cognitive groups could be more sensitive for use in general population samples. Yet, when we applied the stricter cut‐offs proposed by Hancock and Larner (2011) to define cognitive groups in an exploratory manner, the results were very similar to the ones described for this study. In addition, these cut‐offs are less inclusive and could lead to less robust estimates for the relationship estimates between cognitive impairments and the correlates examined. As this is the first study to administer the TYM to a general population sample, we could not calibrate new cut‐offs for use in general population samples in the absence of an additional cognitive screening instrument and/or information on relevant clinical outcomes, for example AD. It is also acknowledged that assigning 5 points to every participant for the last task of the TYM could be a source of bias in terms of the classification obtained. Nonetheless, a sensitivity analysis to examine the classification without assigning 5 points for this question to the study members extracted a classification almost identical to the one reported in the study. Finally, the results presented are based on cross‐sectional assessments, and therefore, only associative but not causative aetiological mechanisms can be inferred, primarily because of possible temporal biases (reverse causality).

### Implications

The proportion of participants classified as having MCI (41%) or SCI (8%) using the TYM in this sample is in line with the prevalence rates for MCI and AD reported in previous studies (Bischkopf *et al*., 2002; World Health Organization, 2012). Available literature reports that the respective prevalence rates for mild forms of cognitive dysfunction and AD range between 2% and 56% (Bischkopf *et al*., 2002) and 2% and 8.5%, respectively, for those aged 60 years and over (World Health Organization, 2012).

Moreover, previous studies, which report similar cardiometabolic risk profiles for cognitive impairments, have used different cognitive screening tools or classification criteria to ascertain classifications of AD or MCI, for example the Mini‐mental state examination, the criteria of the joint‐working group of the National Institute of Neurological and Communicative Disorders and Stroke and the Alzheimer's Disease and Related Disorders Association (McKhann *et al*., 1984) and the Diagnostic and Statistical Manual of Mental Disorders (Third Edition Revised) (APA, 1987). Therefore, future research needs to compare the TYM against established cognitive screening instruments and diagnostic tools to validate its potential as a highly reliable cognitive test for use in general population samples.

## Conclusion

This is the first study to utilize the TYM within a population‐based sample. It provides further evidence that cognitive impairment among older adults is associated with a range of sociodemographic and cardiometabolic factors, particularly low occupation‐based socio‐economic status, physical inactivity, motor and lung dysfunction, smoking, positive history of CVD or diabetes, poor overall health, sleep disturbances and higher levels of functional dependence related to abilities to perform everyday activities. The cardiometabolic and sociodemographic correlates of TYM‐defined cognitive groups are almost identical to those extracted using established screening and diagnostic tools. Targeting these risk factors that are modifiable would increase the scope for primary and secondary interventions aiming to reduce the adverse effects of dementia in the older population.

## Conflict of interest

None declared.

Key points

- This is the first study to administer the Test your Memory (TYM) cognitive screening instrument and to examine the sociodemographic and cardiometabolic correlates of TYM‐defined cognitive function groups in a general population sample.
- The strongest correlates of severe cognitive impairments among older adults are low occupation‐based socio‐economic status, physical inactivity, motor and lung dysfunction, smoking, positive history of cardiovascular diseases or diabetes, poor overall health, sleep disturbances and higher levels of functional dependence related to abilities to perform everyday activities.
- The cardiometabolic and sociodemographic correlates of TYM‐defined cognitive groups in this representative sample of older British men are almost identical to those outlined in current epidemiological models using established cognitive screening tests.

## Sponsor's Role

None.
